# Fecalith Causing Intestinal Obstruction in a Patient with Seckel Syndrome

**Published:** 2014-05-21

**Authors:** Sevgi Buyukbese Sarsu, Burcu Belen, Suleyman Cuneyt Karakus, Naim Koku

**Affiliations:** 1 Department of Pediatric Surgery, Gaziantep Children’s Hospital, 27560, Gaziantep, Turkey;; 2 Department of Pediatric Hematology and Oncology, Gaziantep Children’s Hospital, 27560, Gaziantep, Turkey.;

A 4-year-old girl with SS was admitted with disten-sion of the abdomen, fever, bilious vomiting and non-passage of feces. On the physical examination, a mass with a diameter of 2 cm x 3 cm was palpated at umbilical region. Following the digital rectal exam-ination, a large quantity of feces passed and mass was no more palpable. Plain abdominal x-ray showed dilated small intestinal loops with multiple air-fluid level and a calcified mass in the pelvis (Fig. 1). The mass was confirmed in the computed tomography of the abdomen (Fig. 2). Complete blood count, serum alpha fetoprotein (AFP), carcinoembryonic antigen (CEA) and ferritin levels were normal. Laparotomy was planned for the intra-abdominal mass but it turned out to be impacted fecoloma which was manually removed following rectal irrigation without the need of surgery under general anesthesia.

**Figure:**
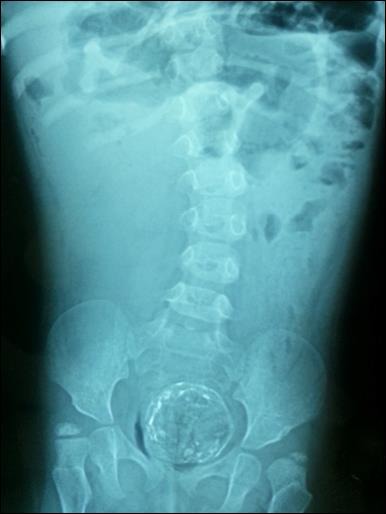
Figure 1: Plain abdominal X-rays showing a calcified mass in the pelvis.

**Figure:**
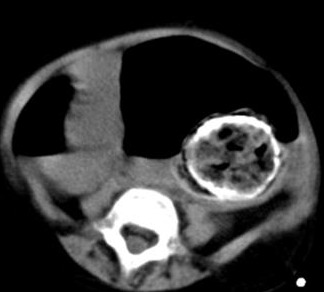
Figure 2: Abdomen computed tomography showed the mass.

## DISCUSSION

Helmut Seckel described Seckel syndrome (SS) which is an autosomal recessive disorder and characterized by cleft lip and palate, club foot, scoliosis, gastrointestinal malformations, and multiple skeletal malformations.[1,2] Other accompanying anomalies are severe microcephaly, craniofacial dysmorphism with characteristic bird headed appearance, promi-nent beaked triangular nose, micrognathia, variable mental retardation, low birth weight, severe intrauter-ine and postnatal growth retardation. Carcinogenesis is usually detected in patients with growth dysregula-tion. Pathogenesis of Seckel syndrome is primarily based on marked growth impairments. There are chromosomal common fragile sites in SS patients making them prone to malignancies.[1-3] 

Intestinal obstruction due fecalith is mainly observed in patients with chronic diseases. In patients with SS, physical disabilities based on central nervous systems abnormalities may be responsible for constipation.[4] Intestinal obstructions due to accumulation of a big fecalith is uncommon. Laparotomy in this patient was decided based on suspicion of tumor. A preoperative rectal irrigation was performed in order to completely rule out a fecal impaction spared the patient from an unnecessary laparotomy.

## Footnotes

**Source of Support:** Nil

**Conflict of Interest:** None declared

